# 中国人肺腺癌细胞系CPA-Yang3及其骨转移细胞的建立

**DOI:** 10.3779/j.issn.1009-3419.2011.02.01

**Published:** 2011-02-20

**Authors:** 顺芳 杨, 梅萍 时, 杰 曹, 建中 苏, 兰香 赵, 贝 雷, 城 常, 建英 陆, 剑定 叶, 文晖 谢

**Affiliations:** 1 200030 上海，上海交通大学附属胸科医院核医学科 Department of Nuclear Medicine, Shanghai Chest Hospital, Shanghai Jiao Tong University, Shanghai 200030, China; 2 200030 上海，上海交通大学附属胸科医院病理科 Department of Pathology, Shanghai Chest Hospital, Shanghai Jiao Tong University, Shanghai 200030, China; 3 200030 上海，上海交通大学附属胸科医院放射科 Department of Pathology; 4 200040 上海，同济大学附属上海第一妇幼保健院病理科 Shanghai Prenatal Diagnosis Center, Shanghai First Maternity and Infant Health Hospital, Tongji University, Shanghai 200040, China; 5 200040 上海，同济大学附属上海第一妇幼保健院产前诊断中心 Department of Radiology, Shanghai Chest Hospital, Shanghai Jiao Tong University, Shanghai 200030, China

**Keywords:** 肺肿瘤, 肿瘤细胞, 模型, 裸小鼠, Lung neoplasms, Tumor cell, Model, Nude mouse

## Abstract

**背景与目的:**

肺癌复发和转移仍是世界性医学难题。本研究旨在建立国人肺腺癌转移细胞株，为研究肺癌转移的分子机制提供新颖的实验材料。

**方法:**

本细胞取自首诊双侧淋巴结转移，65岁女性肺腺癌(Ⅲb期)患者的胸水细胞原代培养即获成功。致瘤率实验，细胞生长曲线，染色体核型分析，荧光定量PCR检测，小鼠心室造模，核素骨显像和X片活体成像检测小鼠骨转移，取骨转移灶细胞原代培养，*in vivo*-*in vitro*重复多个cycles筛选骨转移细胞。

**结果:**

传自第4代细胞小鼠致瘤率达100%，经裸鼠左心室种植(1×10^6^细胞/只)后第3周经核素骨显像、X片和病理检测确定骨转移率为：下颌骨100%，肱骨50%，股骨66.7%，脊柱50%，肩胛骨33%。同时伴有多脏器转移，肺(33%)、肝(50%)、肾上腺(17%)、颌下腺(33%)。荧光定量PCR检测*ESM1*、*VEGF-C*、*IL-6*、*IL-8*、*AR*、*SVIL*、*FN1*等基因过表达。将股骨转移细胞经体内外3个cycles筛选获得骨转移细胞CPA-Yang3BM。

**结论:**

新建的CPAYang3是个取自首诊双侧淋巴结转移肺腺癌病人的胸水原代细胞培养成功并经裸鼠心室造模、体内外4个cycles筛选后获得的多发骨转移的国人肺腺癌细胞株。

## Abstract (en)

**Background and objective:**

The recurrence and metastasis of lung cancer is a tough problem worldwide. The aim of this study is to establish a novel Chinese lung adenocarcinoma cell line and its real bone-seeking clone sub-line for exploring the molecular mechanism of lung cancer metastasis.

**Methods:**

The cells came from the pleural effusion of a sixtyfive years old female patient with lung adenocarcinoma and supraclavicular lymph node metastases. The gene expression was detected by real-time quantitative PCR. Intracardiac injection of the cells into nude mice was performed and *in vivo* imaging was obtained by bone scintigraphy and conventional radiography. Bone metastases were determined on bone scintigraphy and then the lesions were resected under deep anesthesia for bone metastasis cancer cell culture. The process was repeated for four cycles to obtain a real bone-seeking clone.

**Results:**

The tumorigenesis rate started at 4th passage in immunodeficient mice via subcutaneously and as well as later passages. Approximately 1×10^6^ cancer cells were injected into left cardiac ventricle of immunodeficient mice resulted bone metastasis sites were successfully revealed by bone scintigraphy and pathological diagnosis, the mandible (100%), scapula (33%), humerus (50%), vertebral column (50%), femur (66.7%) and accompanied invasion with other organs, the adrenal gland (17%), pulmonary (33%), liver (50%), submaxillary gland (33%) in the mice after inoculation two-three weeks. The chromosome karyotype analysis of the cells was subdiploid. Quantitative real-time PCR was used to examined and compared with SPC-A-1 lung adenocarcinoma, *ESM1*, *VEGF-C*, *IL-6*, *IL-8*, *AR*, *SVIL*, *FN1* genes were overexpress. The novel cell was named CPA-Yang3. The femur metastasis cell was repeated *in vivo*-*in vitro*-*in vivo* with three cycles and harvested a real bone metastasis clone. It was named CPA-Yang3BM.

**Conclusion:**

Tne characteristics of novel strain CPAYang3 is a highly metastasis cell line of Chinese lung adenocarcinoma and CPA-Yang3BM is a real bone-seeking clone.

肺癌的复发和转移仍是世界性的医学难题^[1-4]^。由于种种原因，肺癌患者的低龄化和腺癌化似乎有上升趋势且缺乏有效的诊疗方法和手段。许多患者首诊时即发现有多器官转移^[3]^，其中骨转移占30%-70%。由于肺癌骨转移的生物学机制至今尚不明确，临床缺乏有效的治疗方法。因此，研究肺癌骨转移发病机制并探索有效的诊疗方法是国内外学者长期以来的努力方向。尽管随着分子生物学、信号传导途径、蛋白功能研究等基础学科的发展，人类对疾病发病机理的研究手段有了突飞猛进的发展，但由于种种客观限制，该领域的研究仍未有重要突破。

虽然宿主微环境因素，包括破骨细胞以及一些具有生长因子功能的基质蛋白等对肿瘤的转移具有一定的作用^[3, 5, 6]^，但肿瘤细胞自身生物学特性无疑是骨转移发生更为主要而直接的原因。由于肺癌骨转移的发生大多见于晚期的手术禁忌患者，临床标本取材受限，这在很大程度上迟滞了肺癌骨转移诊疗研究的进展。因此，如能建立有效的人肺癌骨转移模型，不仅能打破取材困难的瓶颈，而且所建模型可以通过多代筛选富集，更有效地发现骨转移相关功能基因，进而利用所建模型直接进行干预性治疗研究，其优越性是临床标本所难以企及的。

2008年6月，我们把取自一名首诊肺腺癌Ⅲb期女性患者的胸水建成体外培养的肺腺癌细胞系，命名为CPA-Yang3。现报道如下：

## 1 材料与方法

### 1.1 病史资料

女性患者65岁。"因胸闷气急一月"来我院就诊时体检扪及双侧锁骨上淋巴结转移并收住入院，胸片和胸部CT：左肺块影伴胸水。支气管镜检查：左总支气管下端粘膜充血性水肿，散在分布大量粟粒状结节影且蔓延至上下叶支气管开口处。活检：腺癌。胸水细胞学检测为阴性。

### 1.2 CPA-Yang3建立过程

采集新鲜血性胸水50 mL分别吸出8 mL放入3个细胞培养瓶，每瓶加入2 mL不含胎牛血清的RPMI1640培养液和三抗(青霉素、链霉素和庆大霉素)后摇匀，放入培养温度为37 ℃、CO_2_浓度为5%的德国贺利氏BB16型二氧化碳孵养箱内培养。5天后光镜下观察时发现细胞培养瓶底约有30%的面积已长满细胞。早期细胞形态以贴壁细胞为主，多为大小不等的多边形，核大、核仁2个-3个，可见分裂相，分裂后细胞仍呈贴壁状，细胞异质性明显。即使传至第30代，细胞形态和生长速度不变。冻存后复苏良好。

### 1.3 致瘤率

细胞培养至第7天，对3瓶长满培养瓶底的细胞用胰酶消化，取细胞数约为4×10^6^，用生理盐水洗2遍后制成细胞悬液，浓度为1×10^7^/mL。分别接种在2只免疫缺陷小鼠(BALB/c)皮下，SPF级饲养。60天后仍不致瘤。传至第4代才致瘤且100%。

### 1.4 染色体核型分析

按常规方法制备中期染色体标本，自然干燥。选择分散好的分裂中期染色体进行染色体计数和显微照相、配对以及核型分析。

### 1.5 绘制细胞生长曲线

取第8代、第26代和第42代3组细胞，分别制成3×10^5^/mL的细胞悬液，吸1 mL加入35 mL细胞培养皿；再加入19 mL含10%胎牛血清的DMEM培养液作细胞培养。每组每天3个培养皿，连续8天细胞计数并绘制生长曲线。

### 1.6 定量RT-PCR法测定CPA-Yang3的*ESM1*^[7]^、*VEGF-C*^[8]^、*IL-6*^[9, 10]^、*IL-8*^[11, 12]^、*AR*^[13, 14]^、*SVIL*^[14, 15]^和*FN1*^[16]^基因表达水平

以SPC-A-1国人肺腺癌细胞为基准，GAPDH为内参，CPA-Yang3为待测细胞样本。细胞总RNA经Trizol试剂(GibcoBRL, Carlsbad, USA)抽提后，行RNA逆转录(Promega, San Luis Obispo, CA, USA)。按ESM1、VEGF-C、IL-6、IL-8、AR序列设计引物(表 1)。定量RT-PCR仪ABI Prism 7900 Sequence Detection System (Applied Biosystems, Foster City, CA, USA)使用SYBR Green Mastermix药盒(TaKaRa, Kyoto, Japan)，检测所得到CT值。通过GAPDH均一化处理，我们对目标基因的表达变化进行计算。每一样本进行了三复孔的定量PCR，取平均值后，数据分析采用公式如下：ΔCt=Ct sample-Ct con；ΔΔCt=ΔCt (geneX)-ΔCt(GAPDH)，Power值是通过计算后取结果的平均值^[17]^。

**1:** *ESM1*、*VEGF-C*、*IL-6*、*IL-8*、*AR*、*SVIL*和*FN1*的PCR引物 Primers for real-time PCR

Gene name	Forward primer	Reverse primer
*ESM1*	GAAGAGCGTCTTGCTGCTGA	ACACTTCATGCCATCCATGC
*VEGF-C*	GCCAACCTCAACTCAAGGAC	CCCACATCTGTAGACGGACA
*IL-6*	AGAGGCACTGGCAGAAAACA	TGCAGGAACTGGATCAGGAC
*IL-8*	CTCTTGGCAGCCTTCCTGAT	ACAACCCTCTGCACCCAGTT
*AR*	GCATCTGAGTCCAGGGGAAC	TCTCGCCTTCTAGCCCTTTG
*SVIL*	CATCCTGGACGGAGTGAACG	TCTCCCTTCTGGCGACTTCC
*FN1*	TGCCCCACTCTCGGAATTC	CGCAGCAACAACTTCCAGGT

### 1.7 裸鼠心内注射造模

实验动物均采用BALB/c裸鼠，8周龄-10周龄，体重18 g-20 g，雄性。由上海市肿瘤研究所提供，并在SPF环境下饲养。

### 1.8 放射性核素荷瘤小鼠活体成像^[18, 19]^

将第4代瘤细胞制成浓度为1×10^7^/mL的细胞悬液种植8只裸鼠左心室，接种量为1×10^6^/只。两周后每周作核素和X线活体成像。每只小鼠尾静脉注射骨显像剂Tc-99m MDP 111MBq，体积0.1 mL，5 h-6 h后分别在Sinmens Multi-spect(Siemens Medical Systems, Inc., Hoffman Estates, Ⅱ., USA)行平面显像和在GE Hawkeye4 Infinia Functional Imaging Scanner(GE Medical Systems, Inc., Waukesha, USA)行微孔针孔显像。平面显像矩阵为256×256，Zoom为2.67。采集计数为(300-500) K/帧；微孔针孔显像矩阵为1024×1024，Zoom为1。采集体位为前后位、后前位、左侧位和右侧位。传统人体X线摄片机(Philips Optimus Bucky Diagnost TS, Philips Healthcare, The Netherlands)行小鼠全身骨骼X线摄片(CR)。将裸鼠麻醉后俯卧位固定，X线胶片置于裸鼠下方。摄片条件：40 kV，2 mA，3 s，28 cmH^[18]^。发现放射性分布异常的骨组织后做好记录，以便无痛处死小鼠后将可疑骨转移灶取出做病理和转移细胞的体外培养。

### 1.9 病理检测和骨转移率

每只在左心室种植CPA-Yang3细胞3周-5周间经核素骨显像、X线检测且称重后麻醉处死，每只鼠统一取股骨、肱骨、胸腰椎和下颌骨共7块骨组织做病理检测，其它部位的骨转移根据核素骨显像决定。将取下的骨组织10%甲醛固定，石蜡包埋，切片，HE染色，光镜下观察裸鼠肿瘤转移情况。然后计算骨转移率。其它脏器转移在解剖探查时记录。

## 2 结果

### 2.1 细胞形态

在相差倒置显微镜下观察CPA-Yang3细胞生长情况。从第二代细胞的相差显微镜图可见细胞呈贴壁生长(图 1)。

**1:**
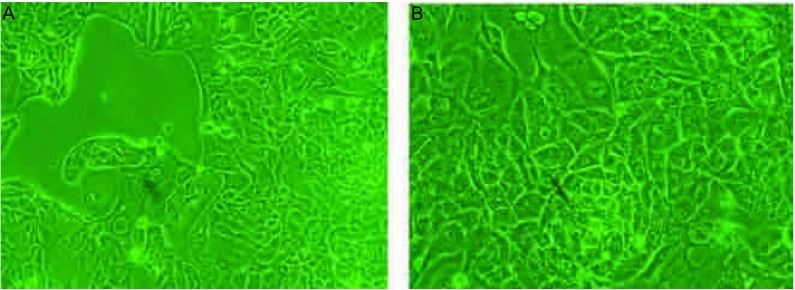
第二代CPA-Yang3细胞相差显微镜图(A：×100；B：×200) The CPA-Yang3 cells in second passage under the contrast phase microscope (A: ×100; B: ×200)

### 2.2 致瘤率

裸鼠皮下种植1-3代细胞均不致瘤，传至第4代后100%致瘤。

### 2.3 CPA-Yang3细胞生长曲线

取第8代、26代和42代细胞分别制成2×10^5^/mL细胞悬液，吸入35 mL细胞培养皿培养；每两天计数，连续8天，可见第2-4天为指数增殖期。第8代、26代和42代细胞的群体倍增时间分别为24.25 h、30.44 h和29.03 h(图 2)。

**2:**
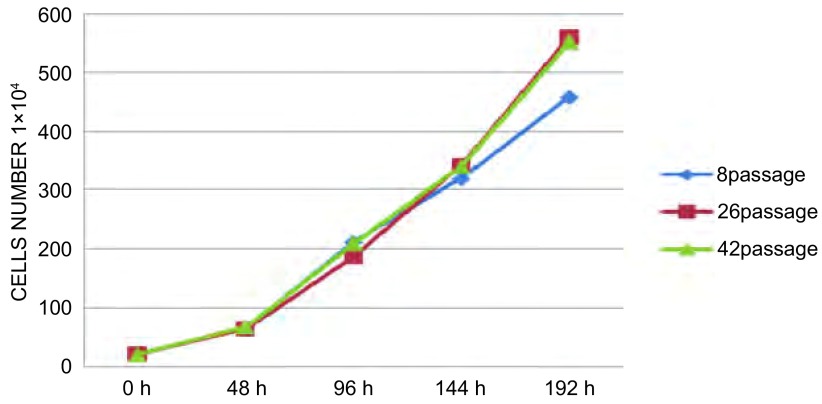
CPA-Yang3细胞生长曲线 The cell growth curve of CPA-Yang

### 2.4 染色体分析

取第8代CPA-Yang3的70个中期分裂相细胞的染色体进行分析，各细胞内染色体数目分布在35-44之间。染色体条数增加的前二位是：第2号和并列第二的1、8、23号；第19、13、14、15号减少；Y缺失。CPAYang3细胞的染色体核型分析图(图 3)。

**3:**
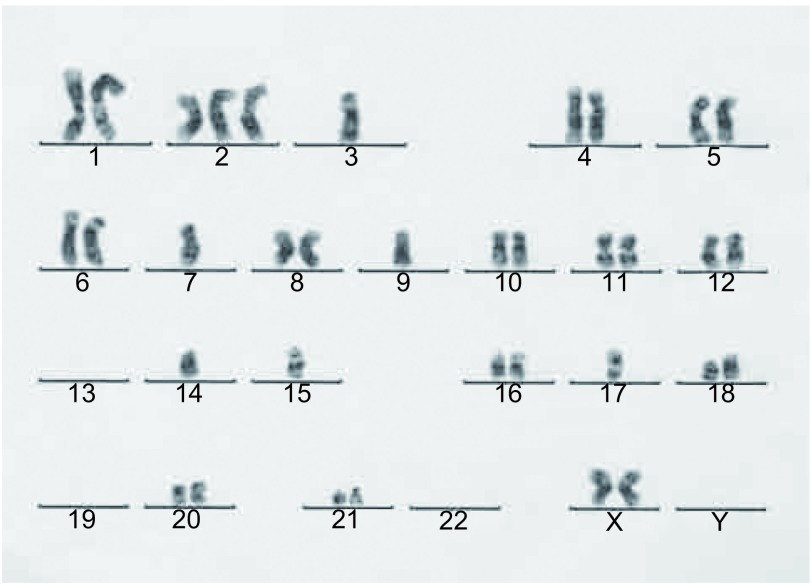
染色体核型分析图 Chromosomal instability in CPA-Yang3

### 2.5 *ESM1*、*VEGF-C*、*IL-6*、*IL-8*、*AR*、*SVIL*和*FN1*基因表

达水平RT-PCR法对国人肺腺癌细胞CPA-Yang3和SPC-A-1进行*ESM1*、*VEGF-C*、*IL-6*、*IL-8*、*AR*、*SVIL*和*FN1*基因表达水平的比较(图 4)。

**4:**
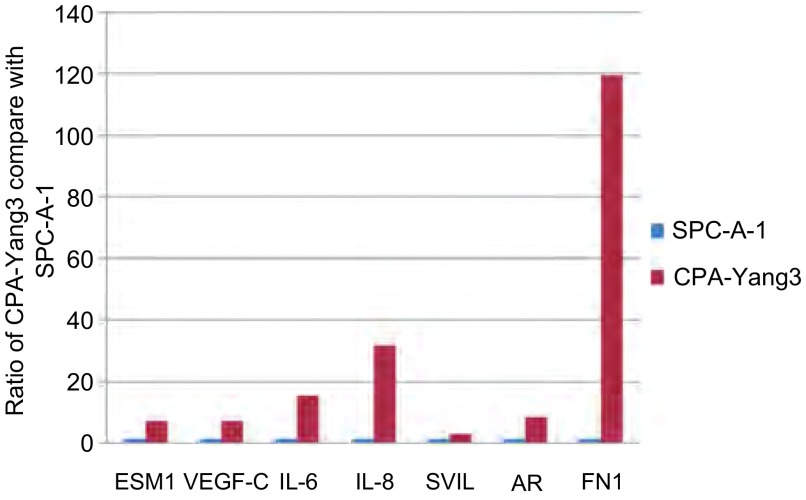
RT-PCR法比较CPA-Yang3与SPC-A-1部分基因*ESM1*、*VEGF-c*、*IL-6*、*IL-8*、*AR*、*SVIL*和*FN1*的表达水平 Real-time PCR was performed on CPA-Yang3 compare with SPC-A-1 to evaluate the expression changes of genes

### 2.6 CPA-Yang3是个除骨转移以外具有肝、肺、颌下腺和肾上腺转移的细胞株

第4代亲代细胞经小鼠体内诱导后给每只裸鼠左心室分别接种1×10^6^/0.1 mL细胞悬液，四周经自创微孔针孔活体成像方法能检出70%骨转移；解剖时肉眼发现有50%肝转移；33%肺和颌下腺转移；17%肾上腺转移。取膝关节转移灶培养骨转移细胞成功扩增后再给裸鼠左心室注射三周后行核素骨显像和X线拍片，80%的鼠在第2个cycle后被核素骨显像检出骨转移灶而不能被X线检出。检出的骨转移灶主要集中在：下颌骨、膝关节、肱骨、脊柱和肩胛骨。还见颌下腺转移。第3个cycle小鼠左心室接种70万骨转移细胞，存活期减至25天-28天。检出的转移灶同前。第4个cycle后小鼠骨转移同前且无其它脏器转移(肝、肺、肾上腺、颌下腺)，因此命名为CPA-Yang3BM。检出的骨转移灶主要集中在：下颌骨、脊柱、膝关节、肱骨、肩胛骨(图 5)。将核素微孔针孔骨显像示小鼠骨转移的同一小鼠拍X片后发现有明显转移的部位仅限于肩胛骨下角约5 mm^3^大小的转移灶(图 6，图 7)。骨转移细胞见图 8。

**5:**
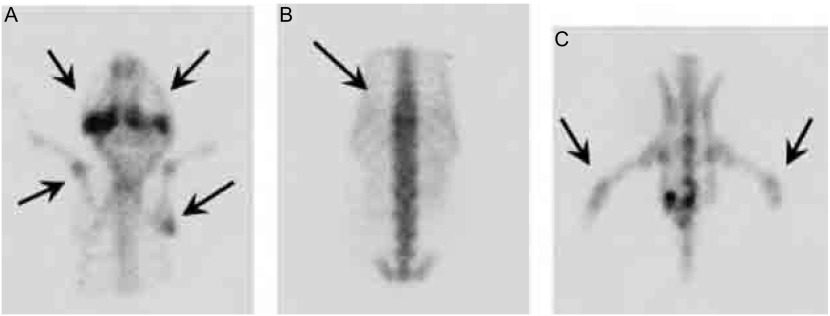
微孔针孔核素骨显像检测小鼠骨转移（后前位）。A：箭头所指左右下颌骨和左肱骨、右肩胛骨转移；B：上箭头示胸椎转移；C：箭头所指两膝关节骨转移。 The pinhole bone scintigraphy detected bone metastasis *in vivo* mice. A: Arrows show jaw bones, left humerus and right scapula metastases; B: Arrows show thoracic vertebrae metastasis; C: Arrows show knee joints metastases.

**6:**
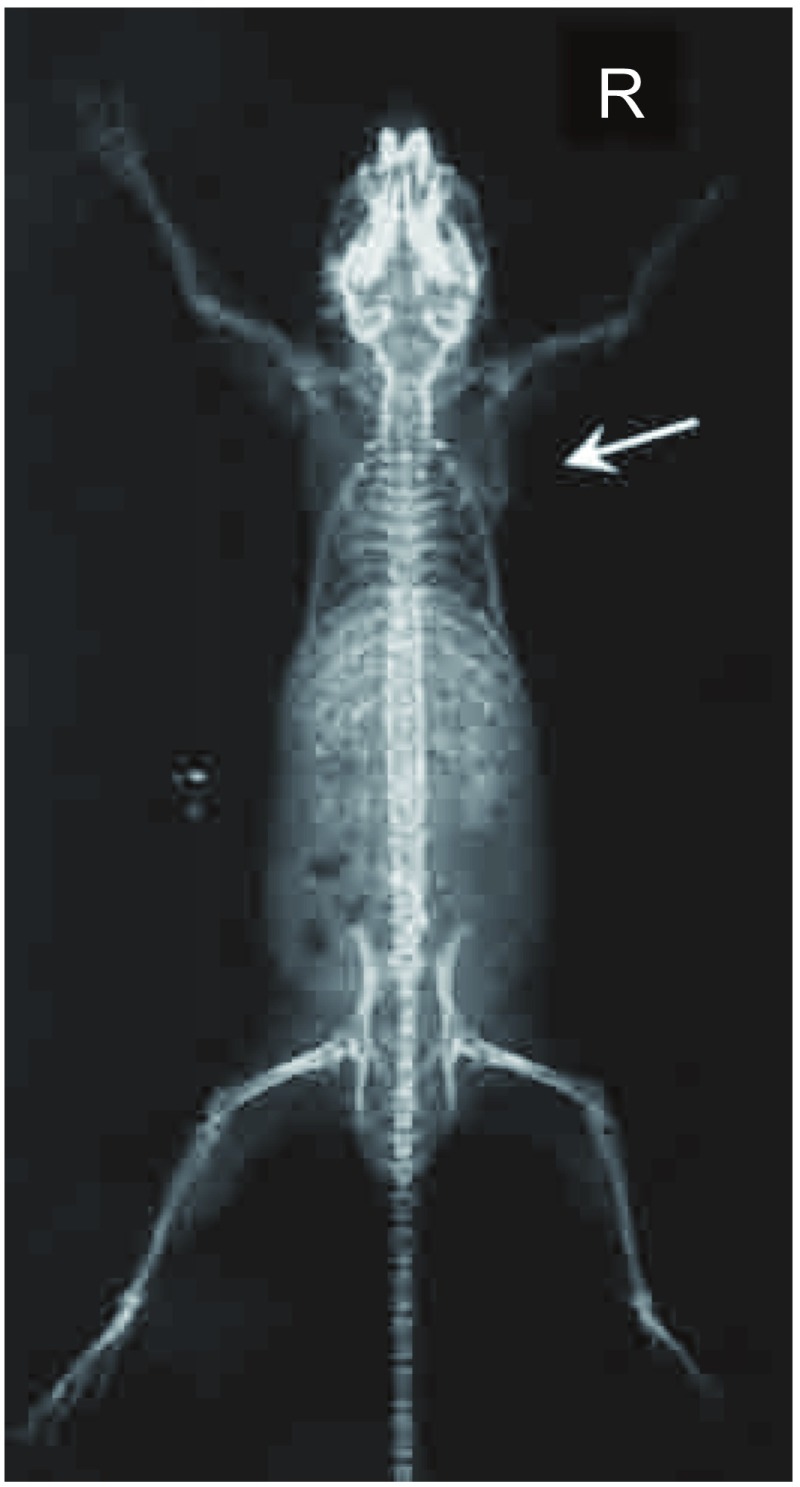
图 5鼠的X线片（后前位） The X ray of Fig 5 mouse

**7:**
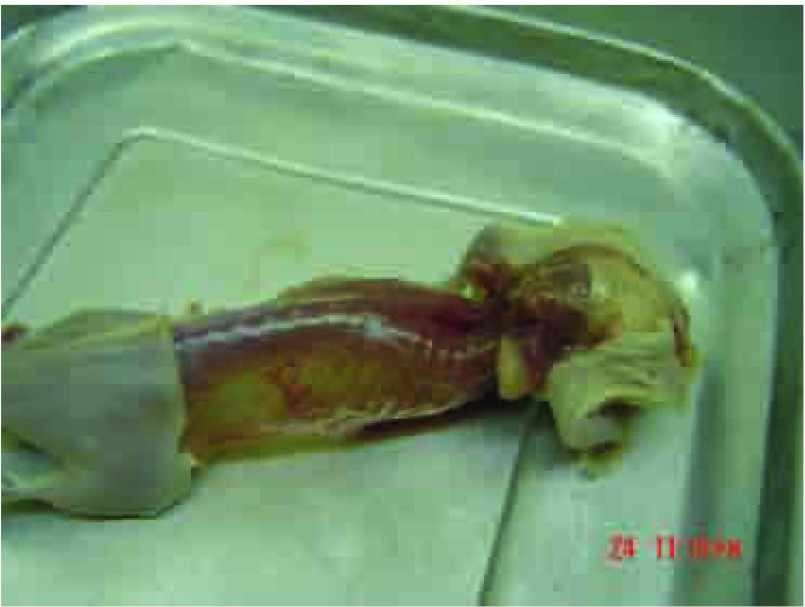
图 5鼠在核素骨显像、X线拍片后解剖时照片 The photo of Fig 5 mouse was bled and then sacrificed after bone scan and X ray detection

**8:**
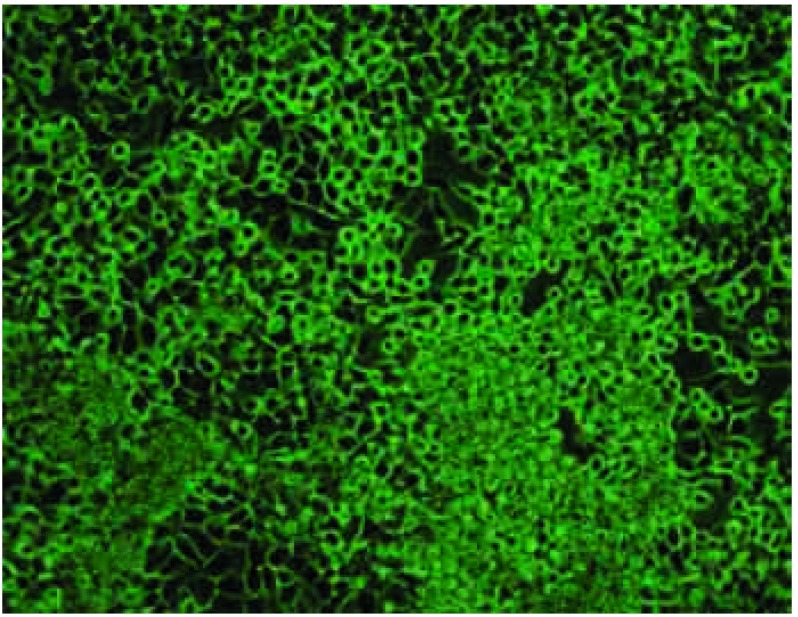
高骨转移亚代细胞CPA-Yang3BM（×100）相差显微镜图 Bone-seeking clone CPA-Yang3BM under the contrast phase microscope (×100)

### 2.7 病理

荷瘤CPA-Yang3小鼠的多脏器转移病理(图 9)

**9:**
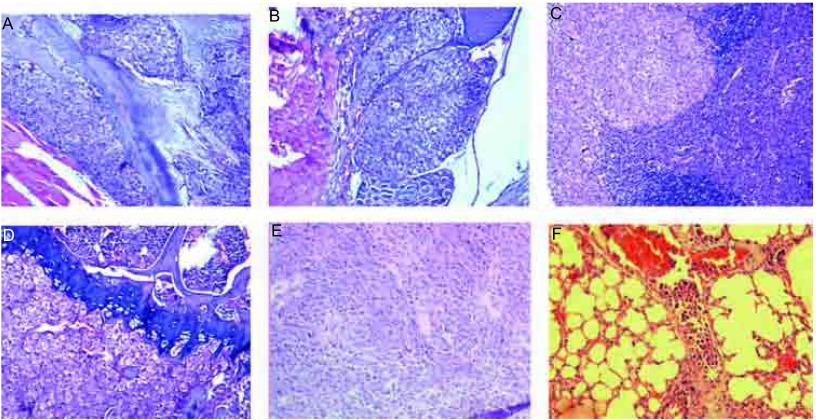
CPA-Yang3经左心室接种后引发小鼠多脏器转移病理(HE, ×100)。A：肩胛骨转移；B：胸椎转移；C：颌下腺转移；D：股骨转移；E：肾上腺转移；F：肺转移。 Histological features of multi-organ metastases in the mouse with CPA-Yang3 (HE, ×100). A: scapula metastasis; B: thoracic vertebrae metastasis; C: submaxillary gland metastasis; D: femur metastasis; E: adrenal metastasis; F: lung metastasis.

## 3 讨论

肺癌的复发和转移仍是肺癌诊治的世界性医学难题，尤其是向特定器官进行靶向转移的分子机理并不清楚。我国学者在肺癌转移细胞系的建立方面做了有益的尝试^[20]^，如部分癌细胞能否专一性很强地向某组织定向转移而不迁徙着床到其它脏器包括原发脏器的争论，其答案基本上是被否定的。但是，新建的国人肺腺癌细胞系中有一些细胞株就具有此特性，CPA-Yang1^[21]^的亲代细胞就具有仅向骨骼转移的特征。CPA-Yang3从开始多器官转移，经过*in vivo*-*in vitro*-*in vivo*四个体内外筛选后得到一株纯骨转移的国人肺腺癌细胞系。但是，国人肺腺癌高转移细胞株SPC-A-1BM经体内外筛选8个cycles后，仍不是一株具有骨转移而无其它脏器转移特性的细胞株。CPAYang2^[22]^传至第11代后方可表现多器官转移特性。归根到底，肿瘤细胞能否专一地向某脏器靶向转移还是由本身的生物学特性所决定的。但要找到这些向特定器官或组织转移的靶标而且对它们的转移机制进行具有说服力的详细阐述说明，这需要付出更多的努力，也是我们正在做的工作。

前期研究^[18, 19, 23]^将不同cycles获得的骨转移细胞SPCA- 1BM和其亲代细胞SPC-A-1杂交，通过cDNA和全基因组的Illumina芯片实验筛选出部分骨转移相关基因。经RT-PCR验证确定几个重点关注的基因并已作了报道。其中部分基因在Yang1-Yang3都得到了验证。

Sarrazin等^[7]^提出ESM1能作为一个肿瘤治疗和检测的标志物和靶标。

IL-6是肺癌的一种自分泌生长因子，肺癌骨转移发生溶骨性骨吸收过程中，IL-6作为诱导破骨细胞生成的细胞因子，通过刺激成骨细胞和基质细胞，增加其表面RANKL表达的间接作用方式，发挥其生物学效应^[9-10]^。

Yuan等^[11]^和Masuya等^[12]^发现肺癌组织中IL-8mRNA表达水平与血管生长、p53抑癌基因异常以及巨噬细胞浸润数量密切相关，并且IL-8过表达者易复发和转移，预后较差。

SVIL是个肌动蛋白结合蛋白(亦称监理蛋白)，在Hela、SW480腺癌和A549肺腺癌细胞株中均有表达。AR的激活是由生长和分化中肌细胞的雄激素而定。SVIL作为AR的参与调节者来自骨骼肌cDNA文库。SVIL能增强PC-3前列腺癌细胞内源性AR的靶基因。SVIL是AR的共同调节者能增强AR在肌细胞和其它细胞的转移激活^[13-15]^。

纤连蛋白(FN1)在促进细胞附着、迁移、分化和肿瘤转移中发挥重要作用^[16]^。FN可激活原癌基因*c-fos*和*c-Jun*的活动，从而使FN的表达与肝细胞癌(HCC)的分化、侵入和转移呈高度相关。FN1在国人肺腺癌骨转移细胞(SPC-A-1BM)中呈高表达^[19]^。RT-PCR法检测*ESM1*、*VEGF-C*、*IL-6*、*IL-8*、*AR*、*SVIL*和*FN1*基因表达水平在国人肺腺癌细胞CPA-Yang3中均上调，肺癌骨转移的发生、发展、形成是否与这些基因相关，值得深入研究和探讨。

## 4 结论

新建的中国人肺腺癌细胞CPA-Yang3和它的骨转移细胞系CPA-Yang3BM是个高潜能转移细胞系，经过四个cycles体内外定向筛选获得骨转移细胞株。这对于骨转移机制的研究又多了个良好的实验模型。
